# Quantification of Intrinsically Disordered Proteins: A Problem Not Fully Appreciated

**DOI:** 10.3389/fmolb.2018.00083

**Published:** 2018-09-04

**Authors:** Sara Contreras-Martos, Hung H. Nguyen, Phuong N. Nguyen, Nevena Hristozova, Mauricio Macossay-Castillo, Denes Kovacs, Angela Bekesi, Jesper S. Oemig, Dominique Maes, Kris Pauwels, Peter Tompa, Pierre Lebrun

**Affiliations:** ^1^VIB-VUB Center for Structural Biology, Vlaams Instituut voor Biotechnologie, Brussels, Belgium; ^2^Structural Biology Brussels, Vrije Universiteit Brussel, Brussels, Belgium; ^3^Research Centre for Natural Sciences of the Hungarian Academy of Sciences, Institute of Enzymology, Budapest, Hungary

**Keywords:** protein concentration, error propagation, nanoorange, coomassie brilliant blue, ninhydrin, UV absorbance, elemental analysis, circular dichroism

## Abstract

Protein quantification is essential in a great variety of biochemical assays, yet the inherent systematic errors associated with the concentration determination of intrinsically disordered proteins (IDPs) using classical methods are hardly appreciated. Routinely used assays for protein quantification, such as the Bradford assay or ultraviolet absorbance at 280 nm, usually seriously misestimate the concentrations of IDPs due to their distinct and variable amino acid composition. Therefore, dependable method(s) have to be worked out/adopted for this task. By comparison to elemental analysis as the gold standard, we show through the example of four globular proteins and nine IDPs that the ninhydrin assay and the commercial Qubit^TM^ Protein Assay provide reliable data on IDP quantity. However, as IDPs can show extreme variation in amino acid composition and physical features not necessarily covered by our examples, even these techniques should only be used for IDPs following standardization. The far-reaching implications of these simple observations are demonstrated through two examples: (i) circular dichroism spectrum deconvolution, and (ii) receptor-ligand affinity determination. These actual comparative examples illustrate the potential errors that can be incorporated into the biophysical parameters of IDPs, due to systematic misestimation of their concentration. This leads to inaccurate description of IDP functions.

## Introduction

Exact determination of protein concentrations is central to modeling in biochemistry, enzymology, molecular biophysics and practically all branches of molecular life sciences. Yet, little attention is paid to various sources of error in this endeavor, with the tendency to rely on rapid, routinely used colorimetric methods based on dye binding, such as that of Coomassie Brilliant Blue (CBB) in the Bradford assay (Bradford, 1976), bicinchoninic acid in the BCA assay (Smith et al., 1985), and Folin's phenol reagent in the Lowry assay (Lowry et al., 1951), or directly measuring ultraviolet (UV) absorbance at 280 nm (Abs280). These techniques are used under the inherent assumption of the uniform behavior of the protein of interest, to bovine serum albumin (BSA) used as a general standard for calibration. For folded and globular proteins this has been a reasonable approximation, with only some, yet notable exceptions (e.g., alcohol dehydrogenase, ovalbumin, cytochrome, and IgG; Szollosi et al., 2007).

However, in the case of intrinsically disordered proteins (IDPs) these techniques usually are not accurate and the large systematic errors can be due to inappropriate standard curves. These discrepancies have already been noted before showing a deviation of an order of magnitude in concentration by the Bradford and BCA assays (Szollosi et al., 2007). IDPs do not fold into a well-defined 3D structure, because of their distinct amino acid composition: they are unusually enriched in Gln, Gly, Ser, Lys, Glu, and Pro (disorder-promoting) and depleted in Trp, Tyr, Phe, Cys, Ile, Leu, and Val (order-promoting) amino acids (Romero et al., 2001). Due to this compositional bias, IDPs typically have low molar extinction coefficient at 280 nm and weak binding of CBB, which relies primarily on hydrophobic interactions with aromatic residues, and electrostatic interaction (between its anionic form) with basic amino acid residues, of the protein (Weist et al., 2008). Therefore, their concentration is often underestimated to a great extent, which is further affected by contaminating macromolecules, causing an often unnoticed error that propagates into their quantitative parameters (Szollosi et al., 2007; Georgiou et al., 2008).

To call attention to this aspect of IDP biochemistry we assessed different quantification methods on four globular proteins and nine IDPs (Table 1). Proteins were selected to cover a wide range of characteristics (size, charge, fold, purity, type of function) and four routinely used methods for protein quantification were chosen: Bradford assay (Bradford, 1976), UV absorbance at 280 nm (Layne, 1957), Qubit^TM^ protein assay, and the ninhydrin assay (Starcher, 2001). The consistency of these methods was assessed by comparing the experimentally determined concentrations of all the proteins to an absolute quantification technique, elemental analysis (EA) (Calderon-Celis et al., 2016).

**Table 1:** Proteins used in this study and their quantification.

	**Protein**	**UniProt #**	**FL/frag-ment**	**C/P**	**EA (mg/ml)**	**Bradford (mg/ml)**	**Abs280 Nanodrop**	**Ninhydrin (mg/ml)**	**Qubit (mg/ml)**	**Abs260/Abs280**
Intrinsically disordered proteins	AF1 (activation function 1 of androgen receptor)	P10275	150–485	P	8.61 ± 0.49	4.29 ± 0.91	8.67 ± 0.08	7.41 ± 0.66	9.74 ± 0.62	0.60 ± 0.00
	EM (*T. aestivum* (wheat) Em protein)	P04568	FL	P	4.00 ± 0.27	2.00 ± 0.76	4.01 ± 0.14	3.70 ± 0.54	5.32 ± 0.39	0.78 ± 0.16
	ERD10 (*A. thaliana* early response to dehydration 10)	P42759	FL	P	3.11 ± 0.38	1.77 ± 0.66	4.28 ± 0.32	2.58 ± 0.56	2.19 ± 0.37	1.10 ± 0.05
	ERD14 (*A. thaliana* early response to dehydration 14)	P42763	FL	P	4.56 ± 0.32	1.19 ± 0.44	21.39 ± 0.83	3.78 ± 0.50	3.73 ± 0.33	1.62 ± 0.03
	hCSD1 (human calpastatin domain 1)	P20810	137–277	P	2.25 ± 0.47	0.52 ± 0.21	6.11 ± 0.04	1.98 ± 0.51	1.78 ± 0.34	1.09 ± 0.01
	ID1 (of CREB-binding protein)	Q92793	1–331	P	7.72 ± 0.44	3.21 ± 0.67	8.41 ± 0.52	7.70 ± 0.81	6.18 ± 0.54	1.06 ± 0.03
	ID5 (of CREB-binding protein)	Q92793	2124–2442	P	8.72 ± 0.59	2.33 ± 0.92	8.91 ± 0.12	8.42 ± 0.57	7.09 ± 0.48	1.04 ± 0.01
	α-synuclein	P37840	1–140	P	4.76 ± 0.32	0.22 ± 0.15	4.70 ± 0.14	4.89 ± 0.34	3.38 ± 0.25	0.52 ± 0.01
	β-casein	P02666	FL	C	6.21 ± 0.40	3.45 ± 0.70	8.23 ± 0.06	5.35 ± 0.73	7.08 ± 0.52	0.62 ± 0.01
Globular proteins	DBD (DNA-binding domain of androgen receptor)	P10275_3	551–644	P	7.77 ± 0.46	11.17 ± 1.35	7.80 ± 0.07	6.62 ± 0.67	7.00 ± 0.50	0.61 ± 0.01
	BSA (bovine serum albumin)	P02769	FL	C	9.85 ± 0.63	9.73 ± 1.51	10.11 ± 0.28	8.92 ± 0.64	11.28 ± 0.70	0.59 ± 0.00
	Hemoglobin	P01966 P02070	FL	C	6.91 ± 0.39	7.39 ± 1.12	4.82 ± 0.08	6.66 ± 0.69	6.37 ± 0.49	0.65 ± 0.02
	Hint1 (human Histidine triad nucleotide-binding protein 1)	P49773	FL	P	7.73 ± 0.47	10.21 ± 0.79	9.16 ± 0.11	6.86 ± 0.63	8.17 ± 0.54	1.01 ± 0.01

*Four globular proteins and nine IDPs have been used in this study. They are identified by their UniProt number, and indicated if the it is full protein (full length, FL), or a fragment of a longer proteins (defined by its terminal residues). The proteins were obtained either from a commercial source (C) or purified in house (P), and the mean value (± the standard deviation) of the measured protein concentrations with the four methods applied, as compared to the absolute concentrations measured by elemental analysis (EA), are given. The ratio of Abs260–Abs280 was measured in triplicate and the average value (± the standard deviation) is represented*.

We show that Bradford assay and abs280 show extreme protein-to-protein variations, whereas ninhydrin and Qubit^TM^ can be reliably used following a single cycle of standardization. Through two actual examples, far UV circular dichroism (CD) spectrum deconvolution and protein to protein affinity determination, we also show how seemingly acceptable levels of error propagate in final functional assessment of IDPs. Thus, we aim to raise awareness of the consequence of this simple, yet non-trivial technical issue. We conclude that appropriate measures to resolve this issue are important for bringing the IDP field to its full quantitative maturity.

## Materials and methods

### Proteins

We included 4 globular proteins (as the different quantification techniques were already extensively tested on this type of protein) and 9 IDPs in this study (Table 1), some obtained from commercial sources whereas others were recombinantly expressed and purified in the lab (Table 2). An outline of the in-house purification procedures is reported in Table 2. We obtained the commercially available proteins as follows: β-casein from bovine milk (cat# C6905) and bovine hemoglobin (cat# 51290) were purchased from Sigma, while bovine serum albumin (BSA; cat# E588) was purchased from Amresco Inc.

**Table 2:** The purification and characteristics of the protein samples that are used in this study.

	**Sub domain of**	**Strain**	**Tag**	**Purification 1**	**Purification 2**	**Purification 3**	**Final buffer**	**Quantification method**	**Concentration (mg/mL)**	**% disorder**	**ε_280 nm_ (M^−1^ cm^−1^)**	**Molecular weight**	**References**
AF1	AR	BL21^*^	His_6_ and smt3	Histrap	Histrap	GF S200 16/100	TBS + 0.5 mM TCEP	UV	10.0	51	30,370	33,887	Unpublished
EM	–	BL21	His_6_	Histrap	GF S7516/60	–	PBS	Weight	7.8	100	1,490	12,150	Unpublished
ERD10	–	BL21^*^	NA	Q FF HiTrap	GF s200 16/60	–	PBS	Weight	7.8	100	2,980	29,547	Kovacs et al., 2008
ERD14	–	BL21^*^	NA	DEAE FF HiTrap	GF s200 16/60	–	PBS	Weight	7.3	100	1,490	20,786	Kovacs et al., 2008
hCSD1	calpastatin	BL21-AI	–	DEAE Hitrap	GF S7516/60	–	30 mM MOPS +1 mM TCEP	UV	7.0	100	4,470	14,764	Nguyen et al., 2017
ID1	CBP	BL21^*^	His_6_, N'-terminal	Ni2+-affinity	ionexchange	GF S200 increase	TBS + 0.05 mM TCEP, 5% glycerol	UV	12	99.7	2,980	38,329	Unpublished
ID5	CBP	BL21^*^	His_6_	Nickel	GF S200 26/60	–	PBS	UV	9.0	100	4,470	38,291	Contreras-Martos et al., 2017
α-synuclein	–	BL21	N/A	DEAE Hitrap	GF S75 26/60	–	TBS	UV	5.3	40	5,960	14,460	Huang et al., 2005
β-casein^$^	–	–	–	–	–	–	–	Weight	10.6	38.5	11,460	25,107	-
DBD	AR	BL21^*^	His_6_ smt3	Histrap	Heparin	GF S75 16/60	TBS + 0.5 mM TCEP	UV	10.0	0	7,450	10,905	Unpublished
BSA^$^	–	–	–	–	–	–	–	Weight	10.0	3.9	42,925	69,293	–
Hemoglobin^$^	–	–	–	–	–	–	–	Weight	10.2	0	47,900	62,275	–
Hint1	–	BL21-AI	His_6_	Nickel	GF S200 16/60	–	PBS	UV	9.5	1.6	8,480	15,369	Unpublished

Protein were obtained from either commercial sources (

$ *) or prepared as recombinant proteins in house. Globular proteins are underlined. For in house purified proteins, the table details major steps of the purification pipeline and references to more detailed protocols when appropriate. The protein characteristics like percentage of intrinsic disorder, the molecular extinction coefficient at 280 nm and the molecular weight are also included*.

* *refers to E. coli BL21 Star strain*.

The computational protein characteristics were obtained based on the amino acid sequence from the UniProt database. The disorder content (% of residues predicted to be disordered) of the proteins was predicted based on the protein sequence with IUPRED through the web server http://iupred.enzim.hu/ (Dosztányi et al., 2005a,b). The “long disorder” option of the predictor was used and disorder % of the protein was determined by considering residues with a predicted value equal to or higher than 0.5 as disordered. The calculated extinction coefficient and the molecular weight were obtained through the ProtParam tool on the ExPASy server (Gasteiger et al., 2005).

To demonstrate that the 9 IDPs permit to draw general conclusions, we have calculated several additional features of the proteins, such as size, net charge, pI, hydrophobic amino acid content, etc… (Table S1). The table demonstrates that the IDPs used cover a broad range of the parameter space, and can be considered as broadly representative of the disordered protein class.

### Protein sample preparation and characterization

Each protein was extensively dialyzed (using 3K molecular weight cut off Slide-A-Lyzer^TM^ dialysis cassettes, Thermo Fisher Scientific) at 4°C against phosphate-buffered saline (PBS) 0.5X supplemented with 0.5 mM Tris(2-carboxyethyl)phosphine HCl (TCEP) with 3 buffer changes (every 6 h). A sodium dodecylsulphate polyacrylamide gel electrophoresis (SDS-PAGE) (MiniProtean-2, Bio-Rad) was performed with a precast any kD gel (BioRad) and Tris-glycin running buffer at 200 V for 30 min. The gel was stained overnight with the commercial PageBlue^TM^ protein staining solution (ThermoFisher Scientific) to evaluate the level of purity using a BioRad ChemiDoc XRS+ molecular imager (Laemmli, 1970).

### Absorbance at 280 nm

UV absorbance at 280 nm (Abs280) was measured on a NanoDrop^TM^ ND-1000 (Thermo Fisher Scientific). Each measurement was done in triplicate and a wavelength scan (340–230 nm) was performed to monitor the Abs260/280 ratio of different proteins.

### Bradford assay

The commercial Quick Start™ Bradford Protein Assay (Bio-Rad) solution was used. The titration curve consisted of 100 μl Bradford reagent (Bio-Rad protein assay) added to the protein or 1–10 μg of BSA standard in a flat-bottom 96-well plate. The absorbance of the sample was measured on a BioTek Synergy Mx plate reader (BioSPX) at 595 nm. Each measurement was done in triplicate.

### Ninhydrin assay

The ninhydrin assay was carried out in triplicate as described previously (Starcher, 2001). As protein standard, 100 μl of 1 mg/ml BSA was hydrolyzed in 100 μl of 6 N HCl at 110°C for 24 h in a heat block. After evaporating to dryness, the hydrolysate was re-dissolved in water to yield a 1 mg/ml final solution. The ninhydrin reagent was prepared as follows: 400 mg ninhydrin (2,2-dihydroxyindane-1,3-dione) was dissolved in a mixture of 15 ml ethylene glycol and 5 ml of 4 N sodium acetate buffer (4 N sodium acetate, pH 5.5, adjusted with glacial acetic acid). Then, 500 μl of stannous chloride suspension (100 mg SnCl_2_ in 1,000 μl ethylene glycol mixed well before pipetting) was added while stirring. A volume of 100 μl of ninhydrin reagent was added to 1–10 μg of protein hydrolysate (final concentration of the unknown sample should be around 1 mg/ml) in a flat-bottom microtiter plate that was sealed with aluminum sealer and floated on a boiling water bath for 10 min. The plate was removed with forceps and blotted with paper towels and the absorbance at 575 nm was measured in a Molecular Devices titer plate reader. The proteins are brought to concentrations so that their measured values were around the middle of the standard curve.

### Qubit protein assay

The Invitrogen Qubit Protein Assay kit (Thermo Fisher Scientific) was used. The titration curve consists of 200 μl of Qubit reagent (diluted 200 times into Qubit reagent) added to 0.2–4 μg of BSA in a flat-bottom 96-well plate. Fluorescence was measured using excitation and emission wavelengths of 485 and 585 nm, respectively, with a BioTek plate reader. Each sample was measured in triplicate.

### Elemental analysis

Lyophilized proteins and calibration standard (ammonium sulfate, ≥99.0% ACS grade, Fluka) were measured into a silver sample holder with 0.01 milligram accuracy, in triplicate. Silver cups were folded and analyzed in a Flash EA 112 (Thermo Fisher Scientific) elemental analyzer. Calibration curve was plotted using known ammonium sulfate quantities and nitrogen content of the protein samples were quantified using interpolation.

### Circular dichroism spectroscopy

The far UV CD spectrum (190–260 nm) of hCSD1 in 200 mM sodium phosphate pH 7.4 was measured on a Jasco 715 spectropolarimeter at 25°C in a thermostated cell holder. The hCSD1 sample concentration was 12.0 μM (i.e., 0.18 mg/ml) as determined by the Qubit assay, or 18.7 μM (i.e., 0.28 mg/ml) based on Abs280. The data were collected in a 1 mm optical pathlength quartz cuvette with a scanning speed of 50 nm/min, a response time of 1 s, a spectral bandwidth of 1 nm, and 5 accumulations. The far UV CD spectrum was corrected for the baseline (by subtraction of the CD spectrum of the buffer collected under identical conditions) without any further data processing.

### Deconvolution of CD spectra

The measured ellipticity in function of wavelength (in the wavelength range of 190–250 nm) was used as input into two CD deconvolution servers to derive the secondary structure composition (we report only α-helicity, β-strand content (as the sum of parallel and antiparallel contributions) and random coil conformation (sum of turn and others)). In the case of the BeStSel server (http://bestsel.elte.hu), two options were used as the data were fitted either with or without concentration correction (Micsonai et al., 2015).

In the case of DichroWeb (with a mean residual weight of 105.45 Da for hCSD1) we tested four different reference sets optimized for the 190–240 nm range (set 4, set 7, SP175, and SMP180) with four different options (Selcon3, ContinLL, CDSSTR and K2D) (van Stokkum et al., 1990; Andrade et al., 1993; Sreerama et al., 1999, 2000; Whitmore and Wallace, 2008). The best fits (by lowest normalized root mean square deviation and upon visual inspection of the plot) were obtained (by reference datasets as noted), as follows: Selcon3 (SP175 reference dataset), ContinLL, CDSSTR (reference set 4 for 12 μM and reference set 7 for 18.7 μM), and K2D (Sreerama et al., 2000; Lees et al., 2006; Abdul-Gader et al., 2011).

### Error propagation in the affinity constant

For visualizing the error propagation in the determination of the K_D_ of a protein-protein interaction, we modeled the results of a traditional equilibrium measurement, in which a R (receptor) molecule is titrated with L (ligand), thus forming an RL complex. We assume that the concentration of L is subject to experimental errors, and determine how it affects the K_D_ determined. Calculations were performed with MATLAB (MATLAB Release 2017a, The MathWorks, Inc., Natick, Massachusetts, United States). For the statistical analysis SPSS was used (IBM Corp. Released 2016. IBM SPSS Statistics for Windows, Version 24.0. Armonk, NY: IBM Corp).

## Results and discussion

### Bradford and Abs280 give highly varying results

**Figure 1:**
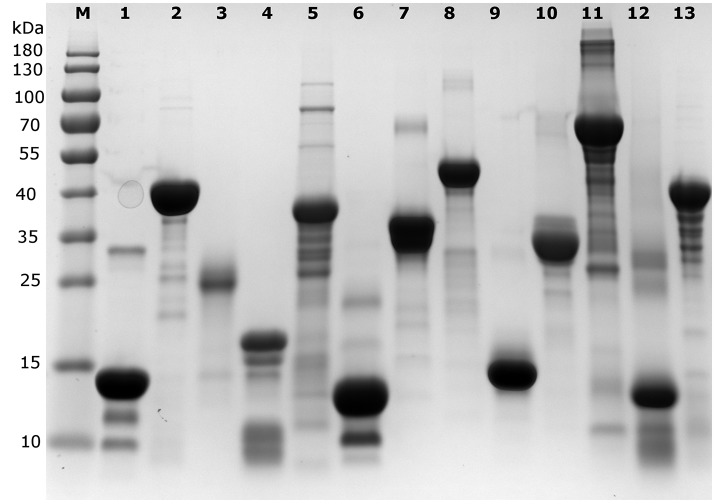
SDS-PAGE analysis of the proteins used in the different quantification assays. An overloaded gel shows only minor impurities in the proteins obtained from a commercial source or purified in house (cf. Table 1). M indicates the lane with marker proteins and their corresponding apparent molecular weight is indicated on the left (in kDa). The protein samples per lane are as follows: 1: Hint1, 2: ID5, 3: hCSD1, 4: α-synuclein, 5: AF1, 6: DBD, 7: ERD14, 8: ERD10, 9: EM, 10: β-casein, 11: BSA, 12: hemoglobin, 13: ID1.

**Figure 2:**
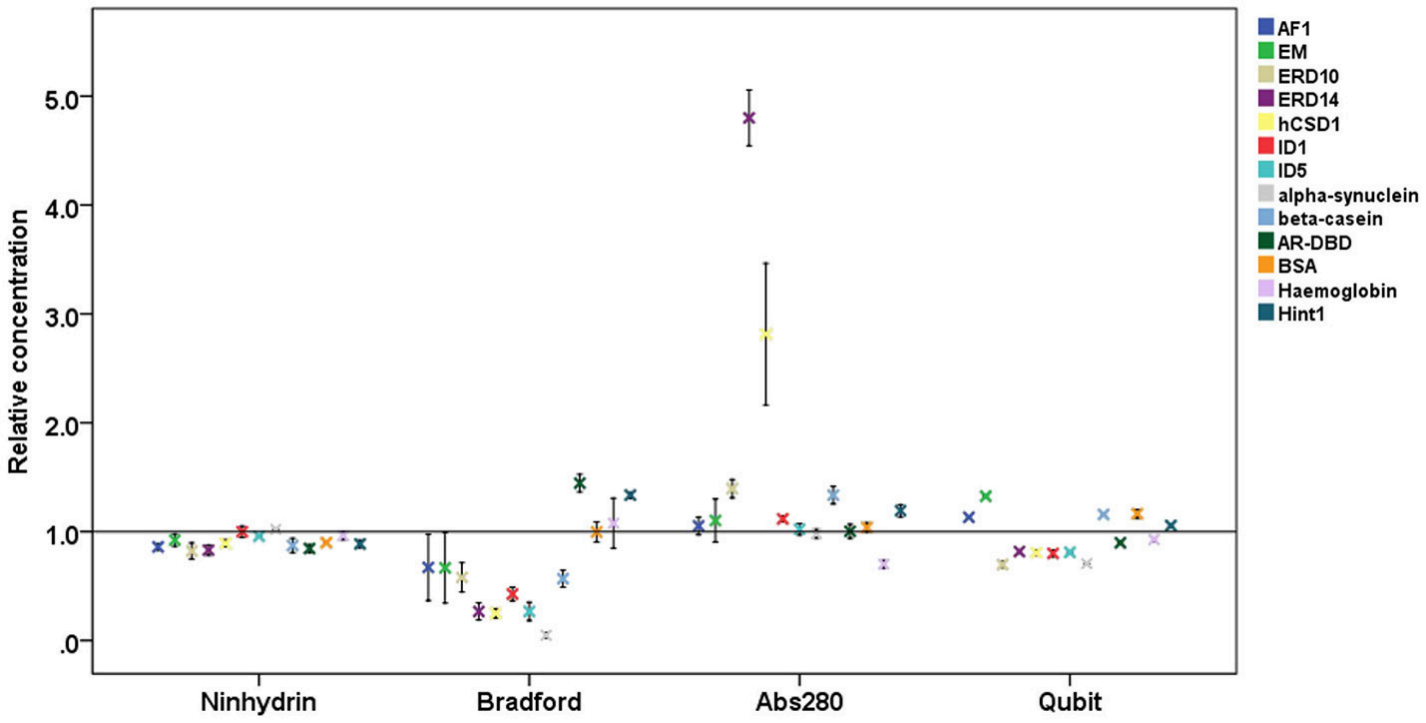
Relative protein concentrations measured by different assays. Results of the concentration measurements by four different methods of 13 proteins (4 globular proteins and 9 IDPs, cf. Table 1), normalized to the absolute concentration measured by elemental analysis. Plots show mean ± *SD* for the four different quantification methods.

**Figure 3:**
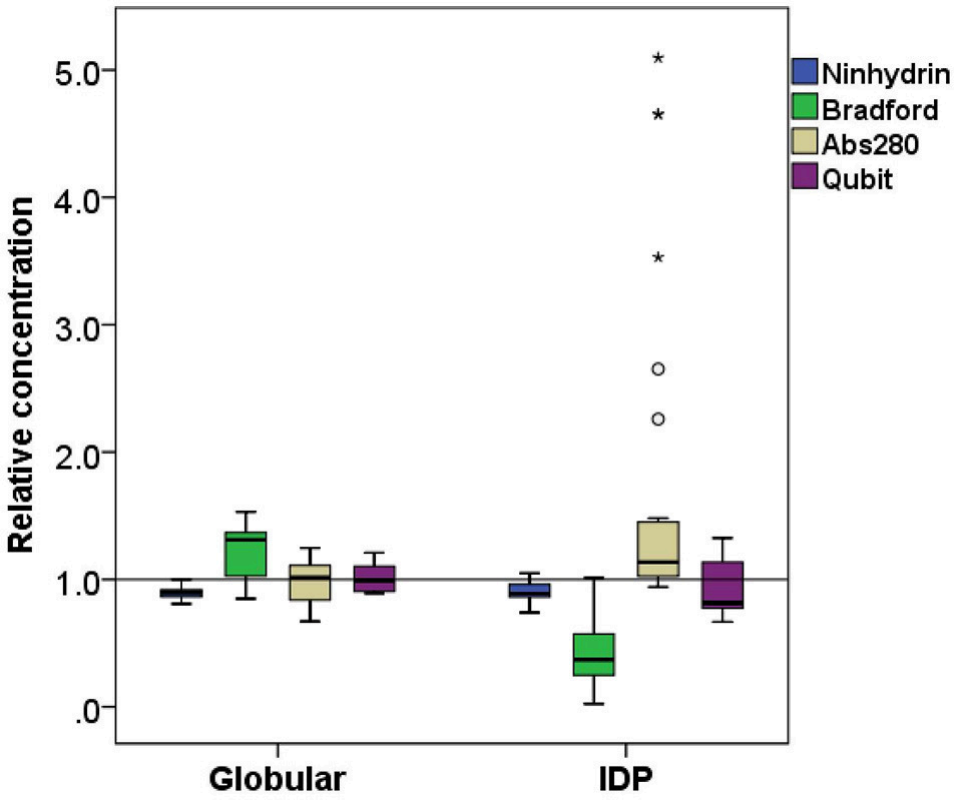
Comparison of relative concentration measurements of folded and disordered proteins. Box plots of the relative concentration of globular and disordered proteins measured by the four methods ninhydrin, Bradford, Abs280, and Qubit.

Each protein (Tables 1, 2) was prepared in a solution of about 10 mg/ml, extensively dialyzed against PBS 0.5X supplemented with 0.5 mM TCEP and its purity was checked on an overloaded SDS-PAGE to visualize the majority of the polypeptidic contaminants (if any) (Figure 1). Their absolute concentration was determined by EA, and it was then measured by all four methods (Table 1, Figure 2). The results are visually represented in Figures 2, 3, which clearly show that different methods perform differently with folded and disordered proteins tested, whereas traditional assays show quite large systematic errors and protein-to-protein variabilities.

On average, the concentration as determined by the Bradford assay is overestimated by 20% for globular proteins, whereas in the case of IDPs it is underestimated by 63% on average (Figure 3). This very large deviation for IDPs has already been reported and clearly posits that the Bradford assay is not adequate for IDPs (Szollosi et al., 2007). By looking into the results in more detail, we observed very large deviations among individual proteins (Figure 2), for example α-synuclein is measured at 5% (0.22 mg/ml instead of 4.76 mg/ml), hCSD1 is measured at 23% (0.52 mg/ml instead of 2.25 mg/ml) whereas ERD14 is measured at 26% (1.19 mg/ml instead of 4.56 mg/ml).

**Figure 4:**
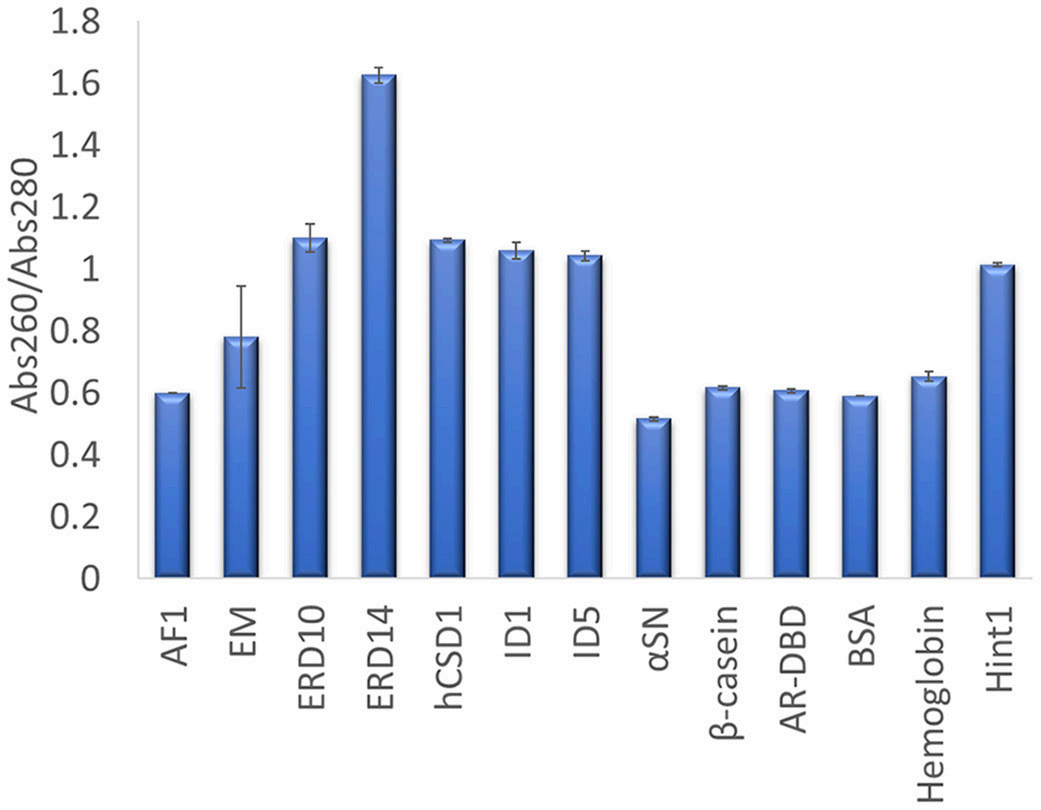
Abs260/Abs280 ratio for each protein determined on a Nanodrop. UV absorbance of each protein was measured in triplicate at 260 and 280 nm, to derive their Abs260/Abs280 ratio. The average value is shown and the error bars represent the standard deviation for each protein sample. A high ratio of ERD14 suggests an inherent nucleic acid contamination.

The other traditional and widely applied method, measuring absorbance at 280 nm, shows even larger deviations (Figures 2, 3). This technique takes into account the absorbance of UV light by tryptophan, tyrosine and cystines, and is thus very sensitive to the amino acid composition of the protein. Due to their paucity of aromatic residues, their extinction coefficient at 280 nm (ϵ_280_) of IDPs is very low, causing great uncertainties at the usual protein concentrations (resulting in very large standard deviations (SDs) and protein-to-protein variations) (Table 2). We have measured the UV absorbance at 280 nm with NanoDrop (Abs280) with a very short optical pathlength (0.2 and 0.5 mm). The Abs280 method is very accurate for globular proteins (mean underestimation of −2%), but fails with most IDPs (+69%, Table 1). By looking at this in more detail, we could make several observations. Firstly, ERD14 has an unusually high Abs260/Abs280 ratio of 1.62 (Figure 4), which suggests that nucleic acids also have a large contribution to the absorbance of the sample at 280, which is erroneously ascribed to the protein, leading to the unrealistic overestimation of its concentration (469%, Figure 2).

Secondly, only 3 out of 9 IDPs (i.e., AF1, α-synuclein and β-casein) display a Abs260/Abs280 ratio of ~0.6 that is expected for pure proteins (Goldfarb et al., 1951; Glasel, 1995). With 5 IDPs (ERD10, ERD14, hCSD1, ID1, and ID5) having Abs260/Abs280 values above 1.0, it becomes clear that this class of proteins is sensitive to nucleic acid contamination, which interferes with a reliable Abs280-based quantification. Interestingly, AF1, α-synuclein and β-casein have the highest ϵ_280_ in our list (Table 2). Thirdly, another observation is that the Abs260/Abs280 ratio for the globular proteins is ~0.6, while Hint1 clearly deviates from this with a value of 1.01. This is likely explained by the fact that Hint1 can bind nucleotides, which can be co-purified and contaminate the protein sample (unpublished observations).

### The ninhydrin and qubit assays are reliable with most IDPs

As opposed to these preceding routine techniques, there are two alternative and less frequently used approaches that perform quite reliably with all the diverse proteins studied.

The Ninhydrin assay is a sensitive technique (Starcher, 2001), and only underestimates all the proteins by 9% (Table 1), with a limited SD (Figure 3). Furthermore, it shows no significant difference between globular proteins and IDPs (Figures 2, 3). This observation can be explained by the fact that following total hydrolysis, the ninhydrin reagent reacts with free amino acids to give a yellow chromophore, i.e., neither the sequence nor the amino acid composition of the protein will affect the assay. The ninhydrin reagent reacts with all the amino acids the same way, with the exception of proline. Although some IDPs are highly enriched in proline (Table S1), previous publications also showed that the amount of proline does not affect the result of the titration (Starcher, 2001). It should be noted, though, that it does underestimate certain proteins (e.g., ERD14, 83%; hCSD1, 88%), thus it should only be used after standardization, i.e., an initial determination of the absolute concentration of the protein and its reaction with the reagent. In addition, one should be aware that the required time for this technique can be a limiting factor, although a protocol of hydrolysis that decreases this time from 24 h even to 2 min, exists (Margolis et al., 1991; Marconi et al., 1995).

The other technique that appears to work reliably with most of the proteins is the Qubit assay, marketed by Thermo Fisher Scientific. The detection method is based on fluorescence developing upon the reaction of the Qubit reagent (NanoOrange) with the protein, probably as originally worked out (Jones et al., 2003), although reaction conditions, such as buffer composition and concentration of reagents, are not specified in the commercial assay. It is extremely accurate with globular proteins (on average, +0.6%, cf. Figure 3) and a bit less for IDPs (on average, −8.5%) (Figure 3). In both cases, the SD is higher than the ninhydrin-based results, as it significantly underestimates (hCSD1, 79%; ID1, 80%) or overestimates (EM, 133%; β-casein, 114%) certain proteins.

### Errors in protein concentration impair biophysical modeling

The measured concentration values and errors of the different methods suggest that for some proteins (especially the folded ones) it is safe to use almost any method for quantification. In contrast, with most IDPs a great care needs to be taken to select the right approach, as there appear deviations of up to an order of magnitude between different methods for certain proteins. The errors will affect the interpretation of subsequent experiments, with potentially severe consequences on quantitative modeling and interpretation. Although one may expect that the error in concentration may simply propagate into the determined chemical/physical parameter and cause a proportionate error in its determination, the problem can actually be aggravated due to non-linear dependence of the parameter on protein concentration. We illustrate this problem through two examples (deconvolution of the CD spectrum of a protein and determination of the affinity of the interaction of two proteins).

#### Case 1: deconvolution of far UV CD spectra

**Figure 5:**
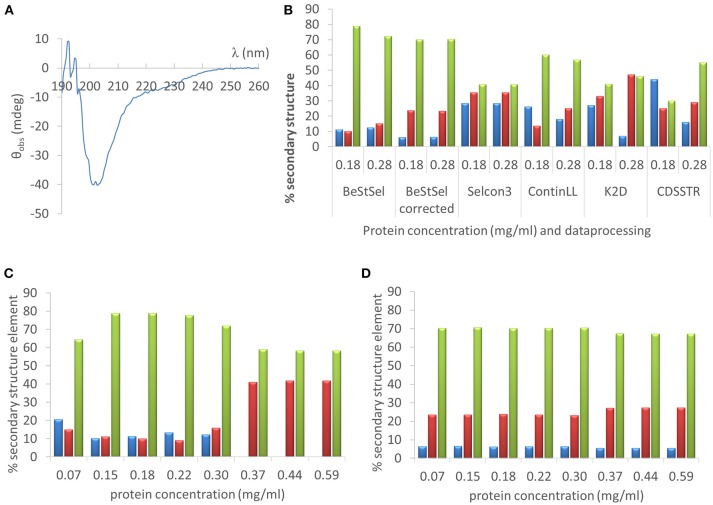
Far UV CD analysis of hCSD1 **(A)** The far UV CD spectrum of hCSD1 corresponds to a prototypical spectrum of a random coil. **(B)** Deconvolution of the CD spectrum of hCSD1 by DichroWeb and BeStSel. The CD spectrum was deconvoluted by assuming two different hCSD1 concentrations (0.18 and 0.28 mg/ml) by either BestSel or through the DichroWeb server. BeStSel was run by two different options (without and with concentration correction), whereas on DichroWeb four different algorithms were used (Selcon3, ContinLL, K2D and CDSSTR). **(C,D)** Deconvolution of the CD spectrum of hCSD1 with BeStSel. The CD spectrum was deconvoluted to yield the % secondary structure composition (α-helix, β-strand and coil) of hCSD1 by BeStSel. The program was run both without **(C)** and with **(D)** the application of “Best” factor correction at a broad, but not unrealistic, range of measured concentrations.

As a first example we deconvoluted the far UV CD spectrum of one of the IDPs studied, hCSD1 (Figure 5A). Estimating the secondary-structure content of a protein from its CD spectrum is probably the most widely used applications of CD. From direct NMR experiments, hCSD1 is shown to be dominated by random coil conformations with transient short α-helices making up about 10% of the structure on the time average (Kiss et al., 2008; Nguyen et al., 2018). As expected, the CD profile of hCSD1 (Figure 5A) is in agreement with the prototypical unstructured polypeptide conformation (Martin and Schilstra, 2008). We used this CD spectrum and 2 experimentally obtained protein concentrations (0.18 mg/ml by Qubit and 0.28 mg/ml by Abs280) with 2 online available deconvolution tools to show the impact of concentration on the fitting of CD data for deriving the secondary-structure content. First, we ran various analysis options on the standard deconvolution server, DichroWeb (Whitmore and Wallace, 2008). The program Selcon3 yielded considerable secondary structure with 28% α-helicity, 35% β-strands and “only” 41% random coil, but it did not reveal any concentration-dependence (Figure 5B). Clearly, with the other analysis programs (i.e., ContinLL, K2D and CDSSTR) within the Dichroweb server, the secondary structure estimation varied with the protein concentration and the deviations are disproportionately (and thus unacceptably) large (Figure 5B.) For example, the α-helix content with K2D goes from 27% (at 0.18 mg/ml) to 7% (at 0.28 mg/ml), whereas with the CDSSTR it goes from 44 to 15% for those same concentrations. In compensation, with CDSSTR, the coil content goes at the same time from 30 to 55%. The secondary structure composition varied less with the concentration when we used a recently developed online tool, BeStSel (Micsonai et al., 2015) (e.g., α-helicity goes from 11 to 13%, β-strand content from 10 to 15%, and random coil from 79 to 72%, for 0.18 and 0.28 mg/ml respectively; Figure 5B). It is of note that there is a considerable difference between the corrected and uncorrected analysis with BeStSel, e.g., the β-strand value goes from 10% (uncorrected) to 23% (corrected). Indeed, in case of uncertainty in the concentration, a “Best” correction factor can be calculated by BeStSel to obtain the lowest NRMSD of the fitted curve, which can still reliably predict the secondary structure content (Micsonai et al., 2015). In general, such a correction seems very important for BeStSel when applied to IDPs, because in this setup it can handle broad concentration variations, such as going from 0.07 to 0.59 mg/ml (Figure 5D). It should be mentioned that the calculation of the Best factor can also be an iterative process that can reveal serious problems with the concentration determination (or optical pathlength). Without applying this correction, there are alarming deviations in deconvolution (e.g., complete disappearance of α-helix at high concentrations, Figure 5C).

It is to be noted that fitting not only depends on the correct protein concentration but also on the optical pathlength, two parameters required to normalize the measured CD data to obtain mean residue ellipticity (Martin and Schilstra, 2008).

In conclusion, an inaccurate concentration determination can have considerable consequences for the secondary structure calculations with state-of-the-art software packages and web-based servers.

#### Case 2: determination of the affinity of an interaction

The error in measured concentration can also have a very large effect on the interpretation of experimental results addressing the dissociation constant (K_D_) of a protein-protein interaction. This might have considerable repercussions on the quantitative modeling and interpretation of biological or physical phenomena. To visualize how an inaccurate concentration determination can affect the measurement of the binding of two proteins, we resort to the case of traditional equilibrium measurements. In such an approach, the simplest 1:1 bimolecular binding reaction is monitored (classically with a spectrometer) whereby a macromolecule R (receptor) binds reversibly with L (ligand) under equilibrium conditions, thus forming an RL complex.

(1) $$\begin{array}{r} R\;+\;L\;⇌\;RL \end{array}$$

Typically, the concentration of R (i.e., [R]) is kept constant throughout the experiment, while increasing amounts of L are added to the sample until saturation is reached. At equilibrium, by definition, the equilibrium dissociation constant K_D_, is equal to the ratio of the concentrations of the free reactants (i.e. [*R*_*F, eq*_] and [*L*_*F, eq*_]) to the concentration of the product (i.e., [*RL*]) at equilibrium (Equation 2).

(2) $$K_{D}=\frac{[R_{F,eq}]\left[L_{F,eq}\right]}{\left[RL\right]}$$

We can measure (accurately or not) the total receptor concentration [R_T_] and at equilibrium it is correct to state that [*R*_*T*_] = [*R*_*F,eq*_] + [*RL*] with [*R*_*F,eq*_] the unbound receptor concentration at equilibrium and [RL] the concentration of the *RL* complex that is formed at equilibrium. In addition, the fractional saturation (the fraction of R that is bound to L) that we designate *f* can be represented by:

(3) $$f=\frac{\left[RL\right]}{\left[R_{T}\right]}$$

This fractional saturation is traditionally followed (by a signal proportional to bound protein) in function of increasing [L]. By combining this information (Equations 2 and 3) the fractional saturation of the receptor can be expressed as:

(4) $$\begin{array}{r} f=\frac{\left[L_{F,eq}\right]}{K_{D}+\left[L_{F,eq}\right]} \end{array}$$

A straightforward calculation shows that for the simple 1:1 bimolecular binding model a fractional error of α on the total ligand concentration (i.e., [*L*_*T*_]_*error*_ = (1+α)[*L*_*T*_]_*exact*_) results in an increase of the fractional error on *K*_*D*_ given by

(5) $$\begin{array}{r} {K_{D}}_{error}=\left(1+\frac{\alpha }{1-f\left[R_{T}\right]/\left[L_{T}\right]}\right){K_{D}}_{exact} \end{array}$$

**Figure 6:**
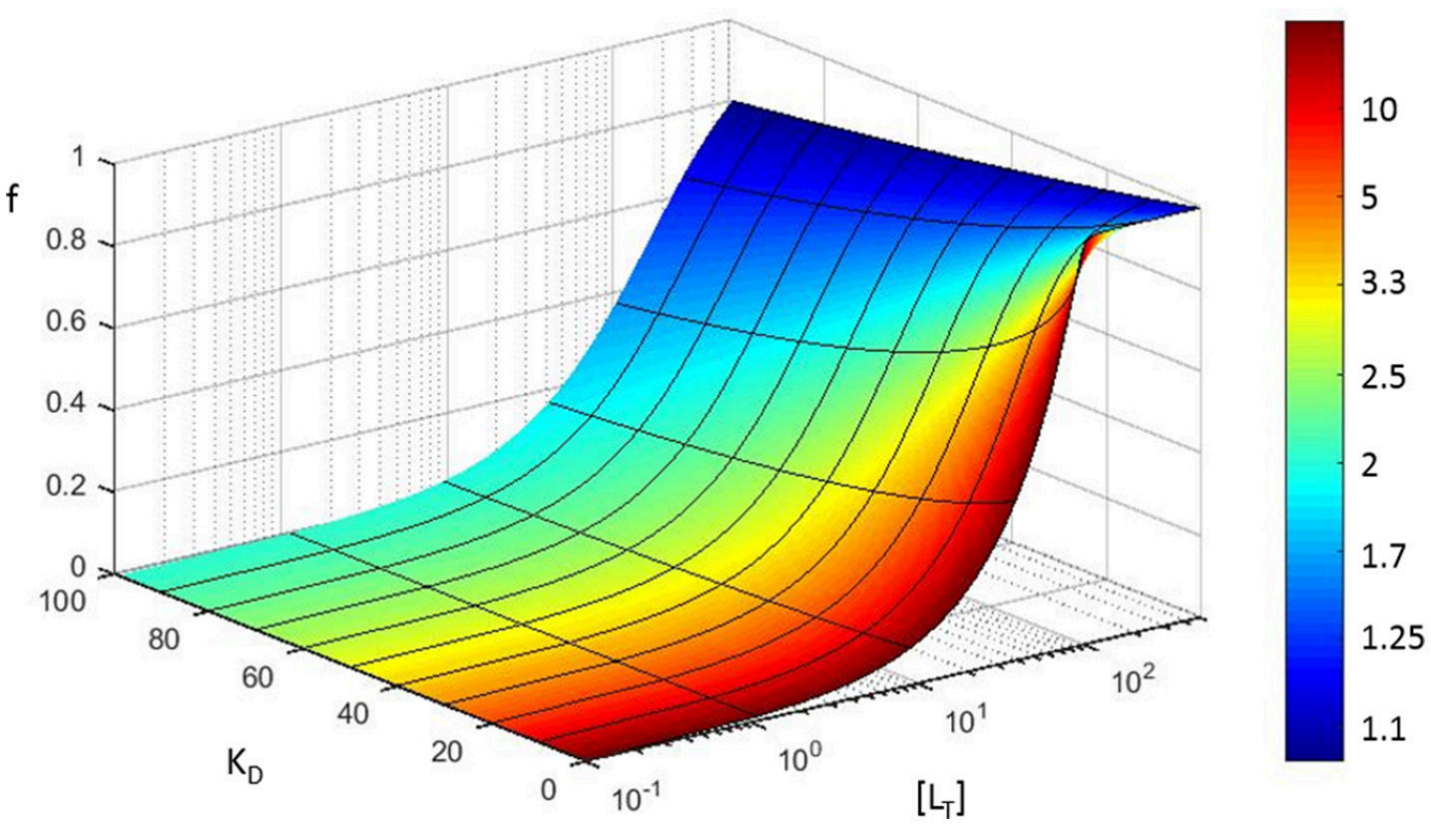
Error propagation in fitting a saturation curve to determine the K_D_ of a protein-protein interaction. Based on a simple model (see section Error propagation in the affinity constant), fractional receptor saturation (f) is shown as a function of the total concentration of ligand ([L_T_]), for different values of the equilibrium dissociation constant (K_D_). The color coding refers to the increase in fractional error on the calculated equilibrium constant (assuming [R_t_] = 100 nM, all concentrations in nM).

This means that the fractional error on *K*_*D*_ is always larger than the fractional error on the concentration. The increase $\frac{1}{1-f\left[R_{T}\right]/\left[L_{T}\right]}$ solely depends on the fractional saturation of the ligand $f\left[R_{T}\right]/\left[L_{T}\right]=\frac{\left[RL\right]}{\left[L_{T}\right]}$. We graphically visualize this error propagation on a 3D plot that shows the fractional receptor saturation *f* in function of the total concentration of ligand for different values of the equilibrium dissociation constant (Figure 6). The color coding in Figure 6 refers to the increase in fractional error on the calculated equilibrium constant.

Note that the fractional error in *K*_*D*_ is a highly non-linear function of the fractional error of the concentration (α), e.g., when 90% of the ligand molecules are in a bound state, the fractional error of *K*_*D*_ is 10 times larger than that of concentration.

## Conclusion

This study, in line with the literature (Szollosi et al., 2007; Georgiou et al., 2008), indicates that the most common assays (UV absorbance and the Bradford assay) are not satisfactory in accuracy for quantifying disordered proteins, as they can result in large systematic errors that reach a factor of 5 in some cases. This error can propagate into derived parameters and can actually be aggravated by the non-linear dependence of parameters on concentration.

Our observations illustrate that due to their biased and highly varied amino acid composition, some IDPs often show unexpected Abs280 values (which can be due to macromolecular contaminants) and CBB, the dye in Bradford assay, hardly interacts with them. The most accurate technique to be sure about the concentration of an IDP is elemental analysis, when the amount of carbon and nitrogen per gram of protein is quantified. Its limitation, of course, is cost, time and the equipment required. Fortunately, there are good alternatives with ninhydrin and Qubit, which are convenient assays with an error range that falls into the acceptable range. Ninhydrin shows a constant deviation of−9,3% with limited SD. Qubit is extremely accurate for globular proteins but the range of its error is increasing with the protein unfolding and its SD is not as good as for ninhydrin. Even if this error is small for the Qubit, the constancy of ninhydrin titration makes it a very powerful tool to quantify proteins with very different characteristics, thus these techniques are recommended for IDP quantification. As demonstrated by comparing amino acid composition parameters and physical features of the IDP sequences tested (Table S1), this conclusion relies on studying IDPs that represent a broad variation in features space. Yet, IDPs may show even more extreme variation in the amino acid composition and physical feature space not necessarily covered by our examples, thus even these methods should be used following a round of standardization based on an absolute method, such as elemental analysis.

## Author contributions

PL, KP, and PT contributed to the conception and design of the work. PL purified Hint1 and performed the ninhydrin assay together with PN. HN purified hCSD1, collected the CD data, performed the SDSPAGE and took care of the protein sample distribution. SC-M purified ID5 and collected the Abs280 data. KP purified α-synuclein and measured the Qubit assay. JO purified AF1 and DBD and measured the Bradford assay. AB purified ID1 and dialyzed all samples. DK purified ERD14, ERD10, EM and prepared β-casein. NH prepared the BSA sample and assisted DK with acquiring the EA data. KP processed the CD data and DM performed the statistical analysis and error propagation modeling. PL and PT performed the interpretation of the data. PL, KP, and PT wrote the paper with contributions of all authors.

### Conflict of interest statement

The authors declare that the research was conducted in the absence of any commercial or financial relationships that could be construed as a potential conflict of interest.
