# Supplementary material for: Quantification of Intrinsically Disordered Proteins: A Problem Not Fully Appreciated
Source: Front Mol Biosci. 2018 Sep 4;5:83. doi: 10.3389/fmolb.2018.00083 (PMC6131523; doi:10.3389/fmolb.2018.00083)
Supplement: Supplementary file 1 [file Table_1.pdf]

Table S1. *Characteristic parameters of the proteins used in this study.*

Nine IDPs and four globular proteins (cf. Table 1) have been used in this study. Their sequences have been analyzed by the ProtParam tool of the ExPaSy server (https://web.expasy.org/protparam/), and a variety of features are given in this table, such as : length, pI, number of negative and positive residues, net charge, % content of hydrophobic amino acids (Ala + Ile + Leu + Val + Trp + Tyr + Phe), proline content and the Grand average of hydropathicity (GRAVY) value as calculated from the sum of hydropathy of all residues of the sequence.

	**Protein**	**UniProt #**	**FL/frag-ment**	**length**	**pI**	**Negative**	**positive**	**net charge**	**hydrophobic amino acids (%)**	**proline (%)**	**GRAVY**
**Intrinsically disordered proteins**	AF1 (activation function 1 of androgen receptor)	P10275	150-485	336	4.57	35	18	-17	32.2	9.2	-0.319
	EM (*T. aestivum* (wheat) Em protein)	P04568	FL	93	5.56	17	16	-1	17.4	1.1	-1.375
	ERD10 (*A. thaliana* early response to dehydration 10)	P42759	FL	260	5.11	65	45	-20	20.9	7.3	-1.348

	ERD14 (*A. thaliana* early response to dehydration 14)	P42763	FL	185	5.4	46	37	-9	23.3	8.6	-1.265
	hCSD1 (human calpastatin domain 1)	P20810	137-277	140	4.85	17	13	-4	27.9	10.3	-0.735
	ID1 (of CREB-binding protein)	Q92793	1-331	331	6.4	15	14	-1	27.2	9.1	-0.398
	ID5 (of CREB-binding protein)	Q92793	2124-2442	319	9.96	9	12	3	20	13.2	-0.936
	α-synuclein	P37840	1-140	140	4.67	4	15	-9	35.8	3.6	-0.403
	β-casein	P02666	FL	224	5.26	23	16	-7	36.6	15.6	-0.154
**Globular proteins**	DBD (DNA-binding domain of androgen receptor)	P10275_3	551-644	94	9.25	9	19	10	29.8	3.2	-0.604
	BSA (bovine serum albumin)	P02769	FL	607	5.82	99	86	-13	36.3	4.6	-0.429

	Hemoglobin	P01966<br>P02070	FL	227	7.39	25	25	0	46	3.5	0.073
	Hint1 (human Histidine triad nucleotide-binding protein 1)	P49773	FL	26	6.43	16	14	-2	37.2	5.6	-0.175
